# The soil microbiome and metabolome in concert shape the flavor profile of ancient tea plants from Laowu mountain region

**DOI:** 10.3389/fpls.2026.1797648

**Published:** 2026-04-23

**Authors:** Qianwen Sha, Qiongfen He, Liang Zeng, Yan Liu, Liyong Luo, Huiting Zhu, Mi Hu, Yunqi Huang, Yamin Wu, Qiaomei Wang, Xiujuan Deng, Lin Tao, Wendi Zhang, Yihu Guan, Wenxia Yuan, Niuniu Shi, Yapeng Li, Ying Qin, Baijuan Wang, Xinghua Wang

**Affiliations:** 1 College of Tea Science, Yunnan Agricultural University, Kunming, China; 2 College of Continuing Education, Yunnan Agricultural University, Kunming, China; 3 Integrative Science Center of Germplasm Creation in Western China (CHONGQING) Science City/College of Food Science, Southwest University, Chongqing, China; 4 Pu’er Wenbang Tea Co., Ltd., Pu’er, China

**Keywords:** ancient tea plants, Laowu Mountain Region, soil metabolome, soil microbiome, tea flavor profile

## Abstract

**Introduction:**

Ancient tea plants from small tea-producing areas in Yunnan possess irreplicable flavor characteristics, yet the mechanisms underlying flavor formation remain unclear.

**Methods:**

This study focused on the core production region of ancient tea plants in the Laowu Mountain Region, including Shahe Village, Hetou Village, and Luojia Village. Differences in tea quality among production regions were analyzed, together with soil physicochemical properties and soil microbial communities, using correlation analysis and amplicon sequencing.

**Results:**

Significant differences in tea quality were observed among different production regions, with catechins, amino acids, and caffeine collectively contributing to these variations. Soil organic carbon, organic matter, and nitrate nitrogen showed significant differences between production regions. Correlation analysis revealed that soil organic carbon was significantly positively correlated with epigallocatechin (EGC) (r > 0.8, *P* < 0.05), while soil nitrate nitrogen and organic matter were significantly negatively correlated with epicatechin (r < -0.8, *P* < 0.05). Amplicon sequencing indicated that the dominant bacterial phyla in the soil included *Chloroflexi*, *Acidobacteriota*, *Proteobacteria*, and *Actinobacteriota*, while the dominant fungal phyla were *Ascomycota*, *Basidiomycota*, and *Mortierellomycota*. Spearman correlation analysis showed that *g:Streptomyces* was negatively correlated with amino acid metabolites but positively correlated with total amino acids (AA) in tea, whereas amino acid metabolites were negatively correlated with AA. Meanwhile, *g:Bacillus* was negatively correlated with gibberellin A7 and GA, but positively correlated with EGC, while gibberellin A7 was positively correlated with GA and negatively correlated with EGC.

**Discussion:**

These results shed new light on the mechanisms by which soil microorganisms and metabolites collaboratively shape the flavor compounds of ancient tea plants, while also providing a basis for the soil ecological management of Yunnan ancient tea plants.

## 1 Introduction

Yunnan Province, one of the origins for *Camellia sinensis (L.)* Kuntze possesses abundance ancient tea plant resources (Wang et al., 2025). Ancient-plant Pu-erh tea (APPT), which is produced from the fresh leaves of ancient tea plants through specific processing techniques, serves as a key driver of the distinctive development of the Yunnan tea industry due to its rarity and unique flavor characteristics (Wang et al., 2023; Fang et al., 2024). Based on their origin places, APPT are classified into several small tea-producing areas such as Laowu Mountain, Laobanzhang, Yiwu, and Bingdao. The vertical climate and complex terrain across different small tea-producing areas have led to significant variations in the quality of APPT from these regions (Li et al., 2024). However, the key driving factors underlying variations in tea quality among small tea-producing areas remain unclear.

Beyond providing essential nutrients, soil serves as the ecological basis for tea plant growth, shaping tea quality through the interplay of microorganisms and metabolites (Doménech-Pascual et al., 2025; Zhou et al., 2025). Soil microorganisms drive nutrient cycling and organic matter decomposition, thereby regulating the accumulation of flavor-related compounds, such as amino acids, phenolics, and volatiles, in tea leaves (Yang et al., 2018; Hector, 2022; Zhao et al., 2025). Simultaneously, microbial-derived soil metabolites reflect these biochemical processes and further modulate the soil microenvironment (Li et al., 2023, Li et al., 2025). Compared to modern, intensively managed plantations, ancient tea gardens maintain a more stable and complex belowground ecosystem (Sugden, 2019). Through long-term evolution, ancient tea plants have adapted to interact with broader microbial communities and utilize deep soil resources, making their metabolism heavily dependent on these specific ecological processes (He et al., 2023). However, while current research on ancient tea largely focuses on climate, topography, and physicochemical factors, our understanding of how soil microorganisms and metabolites jointly shape its unique flavor profile remains limited.

The ancient tea plants in the Laowu Mountain Region boast a long history of growth, minimal human disturbance, excellent soil quality, and superior tea quality. With intact communities of ancient tea plants, this region acts as a representative region for investigating the interactions between soil and ancient tea plants. Based on the plant–soil feedback mechanism, we hypothesized that soil microenvironments across different core production regions of Laowu Mountain shape spatially heterogeneous microbial communities and, in turn, distinct soil metabolite profiles. These soil metabolites serve as key links between the soil microecology and tea plant physiological metabolism by mediating nutrient uptake and signal transduction, thereby driving the selective accumulation of key flavor compounds in tea leaves.

To investigate the influence of soil microorganisms and metabolites from ancient tea plants in Yunnan on tea quality, and to test the proposed hypothesis, this study focuses on the quality differences of ancient tea plants from three core production regions in the Laowu Mountain Region, namely Shahe (SH), Hetou (HT), and Luojia (LJ). It aims to determine the physicochemical properties of fresh leaves and soils of ancient tea plants, and analyze differences among these production regions. Non-targeted metabolomics (LC–MS) and high-throughput sequencing (16S/ITS) were employed to systematically characterize region-specific soil metabolites and signature microbial communities. Furthermore, multi-omics association analysis was conducted to construct a soil microbiome, metabolome, and tea quality interaction network. Finally, we investigated the relationships among soil microorganisms, soil metabolites, and tea quality. In summary, this study aims to reveal the coupled regulatory mechanisms linking soil properties, microorganisms, metabolites, and tea quality in the core ancient tea-growing area of the Laowu Mountain Region. It seeks to elucidate the pivotal role of soil microorganisms and metabolites in tea quality regulation mediated by the soil environment, thereby laying a theoretical foundation for understanding quality formation mechanisms and enabling precise management of small production regions of ancient tea plants in Yunnan.

## 2 Materials and methods

### 2.1 Overview of production regions and sample collection

**Figure 1:**
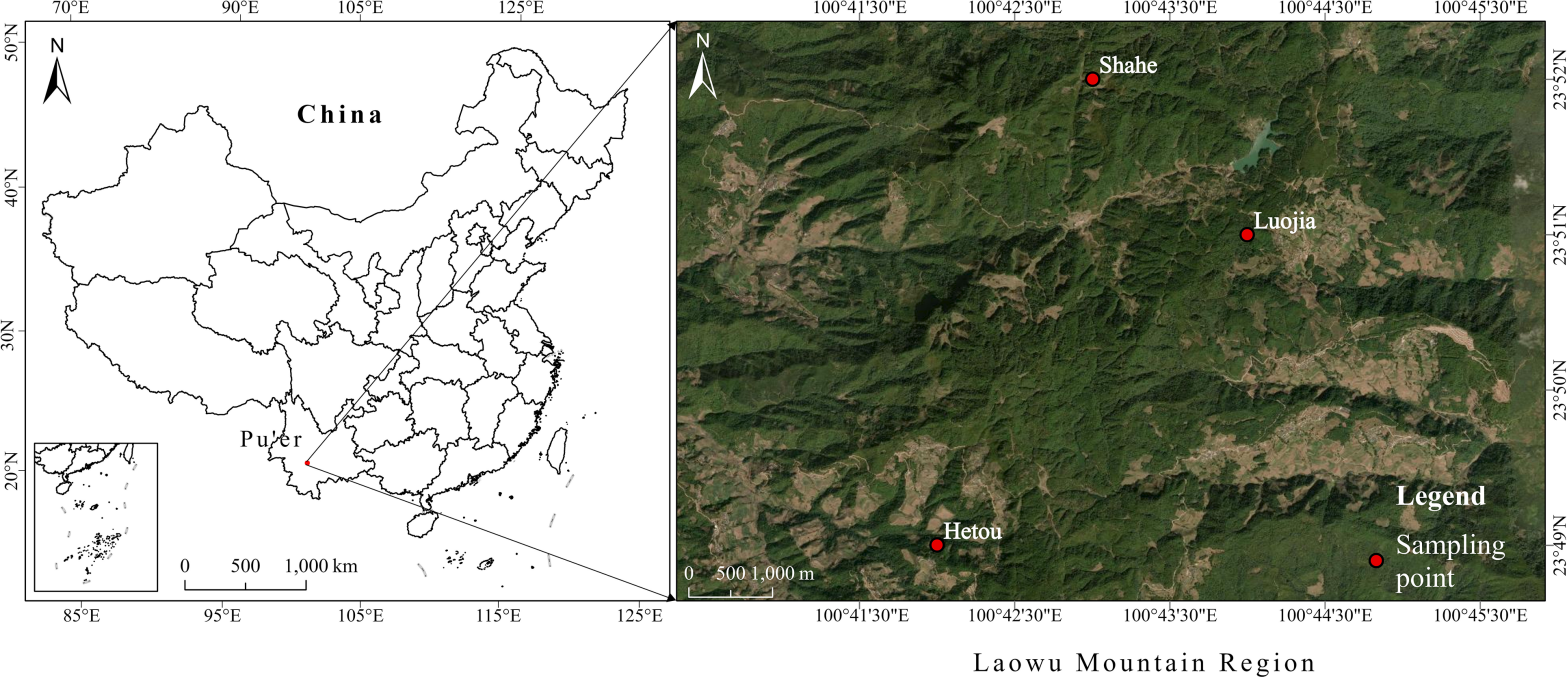
Sampling point information.

Samples were collected from three core production regions of ancient tea plants in the Laowu Mountain Region, namely Shahe (SH), Hetou (HT), and Luojia (LJ) (**Figure 1**), in October 2023. These three regions are geographically close, located at comparable altitudes, and belong to the same tropical monsoon climatic zone. The study area has an annual mean temperature of approximately 14 °C and an annual precipitation of 1390–1502 mm. The dominant soil types in this region are red soil and yellow-brown soil. The tea plant resources in the study area mainly include Tengtiao tea, ancient tea trees, cultivated ancient tea trees, and wild tea communities. Agronomic management practices were broadly similar across the three sampling regions. Specifically, deep tillage was conducted once annually from December to January, and mechanical weeding was carried out once in October, accompanied by manual pruning to remove weak or diseased branches, old leaves, tea fruits, tea flowers, trunk mosses, and parasitic plants. Sheep manure was applied once every three years, usually in December. During tea plant growth, the main pest was stem borers, which were controlled primarily through physical removal during routine pruning and field management, without pesticide application. These comparable environmental and management conditions were intended to minimize the influence of non-soil environmental variation on tea quality.

Based on the terrain characteristics of each production region, five soil samples were collected from each region. For each soil sample, five subsamples were taken from the 0–40 cm soil layer and combined into one composite sample. The sampling points were distributed across the entire production region to ensure representativeness. Each soil sample was divided into two portions: one portion was rapidly frozen in liquid nitrogen and stored at −80 °C, and the other portion was naturally air-dried, sealed, and reserved for subsequent analyses. In addition, three mixed fresh tea leaf samples were collected from each production region. All leaf samples were collected at the same developmental stage, namely one bud with two or three leaves. The samples were cryopreserved using dry ice and then stored at −80 °C for subsequent analyses.

### 2.2 Determination of physicochemical components in tea

The main physicochemical components of tea leaves were measured using spectrophotometry and HPLC. Water extractables, tea polyphenols, and free amino acids were measured using the spectrophotometric method described by Wang et al(T. Wang et al., 2025). Moreover, 11 compounds encompassing catechins, alkaloids, flavonoids, and phenolic acids were isolated and quantified using an Agilent 1200 series HPLC system (Agilent Technologies, Santa Clara, CA, USA) paired with an Agilent Poroshell 120 EC-C18 column (4.6 mm × 100 mm, 2.7 μm).

### 2.3 Determination of soil physicochemical properties

The Soil Physical and Chemical Properties were conducted according to our previous studies (Li et al., 2024). Briefly, soil pH was determined potentiometrically, and electrical conductivity was measured with the electrode method. Soil organic carbon (OC) and organic matter (OM) were quantified via potassium dichromate oxidation spectrophotometry and oil bath-heated potassium dichromate oxidation, respectively. Total phosphorus and available phosphorus (AP) were assayed by alkali fusion-molybdenum antimony colorimetry and sodium bicarbonate leaching-molybdenum antimony colorimetry, respectively. Total potassium (TK) was analyzed using inductively coupled plasma-atomic emission spectroscopy (ICP-AES), while available potassium (AK) was determined via neutral ammonium acetate leaching followed by flame photometry. Ammonium nitrogen (NH4^+^-N) and hydrolyzable nitrogen were measured through leaching-colorimetry and alkaline diffusion, respectively. Soil particle size and specific gravity were determined using the pipette-hydrometer combined method and the density bottle method, respectively.

### 2.4 Metabolomics data collection employing liquid chromatography-MS/MS technique

A 50 mg aliquot of the solid sample was introduced to a 2 mL centrifuge tube with 6 mm grinding beads. Metabolites were extracted with 400 μL of a methanol-water mixture (4:1, v/v) supplemented with 0.02 mg/mL L-2-chlorophenylalanine as internal standard, and the resulting supernatant was obtained via centrifugation for subsequent assays. Quality control samples were pooled for analysis. Samples (3 µL) were separated using an HSS T3 column and analyzed by mass spectrometry. The mobile phase comprised 95% water/5% acetonitrile/0.1% formic acid. The flow rate was 0.40 mL/min, and the column temperature was maintained at 40 °C. The MS was operated in both positive and negative ion modes (m/z 70-1050), 3500/2800 V ion spray, sheath/gas at 40/10 psi, ion source temp 400 °C, CE 20–60 V. The resolution was set to 70,000 for MS1 and 17,500 for MS2.

### 2.5 Metabolite substance identification and analysis

After acquisition, LC-MS data were processed in Progenesis QI (Waters Corporation, Milford, USA) for baseline filtering, peak alignment, and peak integration, generating a data matrix containing retention times, m/z values, and peak intensities. Metabolite annotation was performed by matching MS/MS data against the HMDB database (http://www.hmdb.ca/). Missing values were filtered according to the 80% rule, peak intensities were normalized using sum normalization, and variables with a relative standard deviation (RSD) > 30% in QC samples were excluded. The resulting data were log10-transformed to generate the final analysis matrix. Model stability was evaluated by 7-fold cross-validation. Differential metabolites were screened based on statistical significance after false discovery rate (FDR) correction and fold change, using the criteria of adjusted *P* < 0.05 and FC > 1.2 or FC < 0.8. Identified differential metabolites were subsequently mapped to KEGG pathways for functional annotation (https://www.kegg.jp/kegg/pathway.html).

### 2.6 DNA sequencing and analysis

Microbial genomic DNA was extracted using the E.Z.N.A.^®^ Soil DNA Kit, and DNA quality was assessed by agarose gel electrophoresis and quantified using a NanoDrop 2000 spectrophotometer. The V3–V4 region of the bacterial 16S rRNA gene was amplified using primers 338F/806R, and the fungal ITS region was amplified using primers ITS1F/ITS2R. Libraries were prepared using the NEXTFLEX Rapid DNA-Seq Kit and sequenced on the Illumina NextSeq 2000 platform with paired-end reads (2 × 150 bp). The average sequencing depth was approximately 30,000 reads per sample. Raw sequences were deposited in the NCBI Sequence Read Archive (SRA) under accession number SRP570778. After quality filtering with fastp and read merging with FLASH in QIIME2, chloroplast and mitochondrial sequences were removed, and samples were rarefied to 20,000 reads for downstream analysis. Sequences were clustered into operational taxonomic units (OTUs), which were taxonomically classified against the SILVA database (version 138) using the Naive Bayes classifier, VSEARCH, or BLAST. Functional profiling was predicted using PICRUSt2. Alpha diversity indices (Chao1 and Shannon) were calculated in mothur, with intergroup comparisons performed using the Wilcoxon test. Beta diversity was assessed using Bray–Curtis distance-based principal coordinates analysis (PCoA), and community differences were tested by PERMANOVA. Differentially abundant taxa across taxonomic levels were identified using LEfSe (Linear Discriminant Analysis Effect Size) with thresholds of LDA > 2 and P < 0.05 (Li et al., 2024).

## 3 Results

### 3.1 Variations in quality characteristics of teas from three core production regions

**Figure 2:**
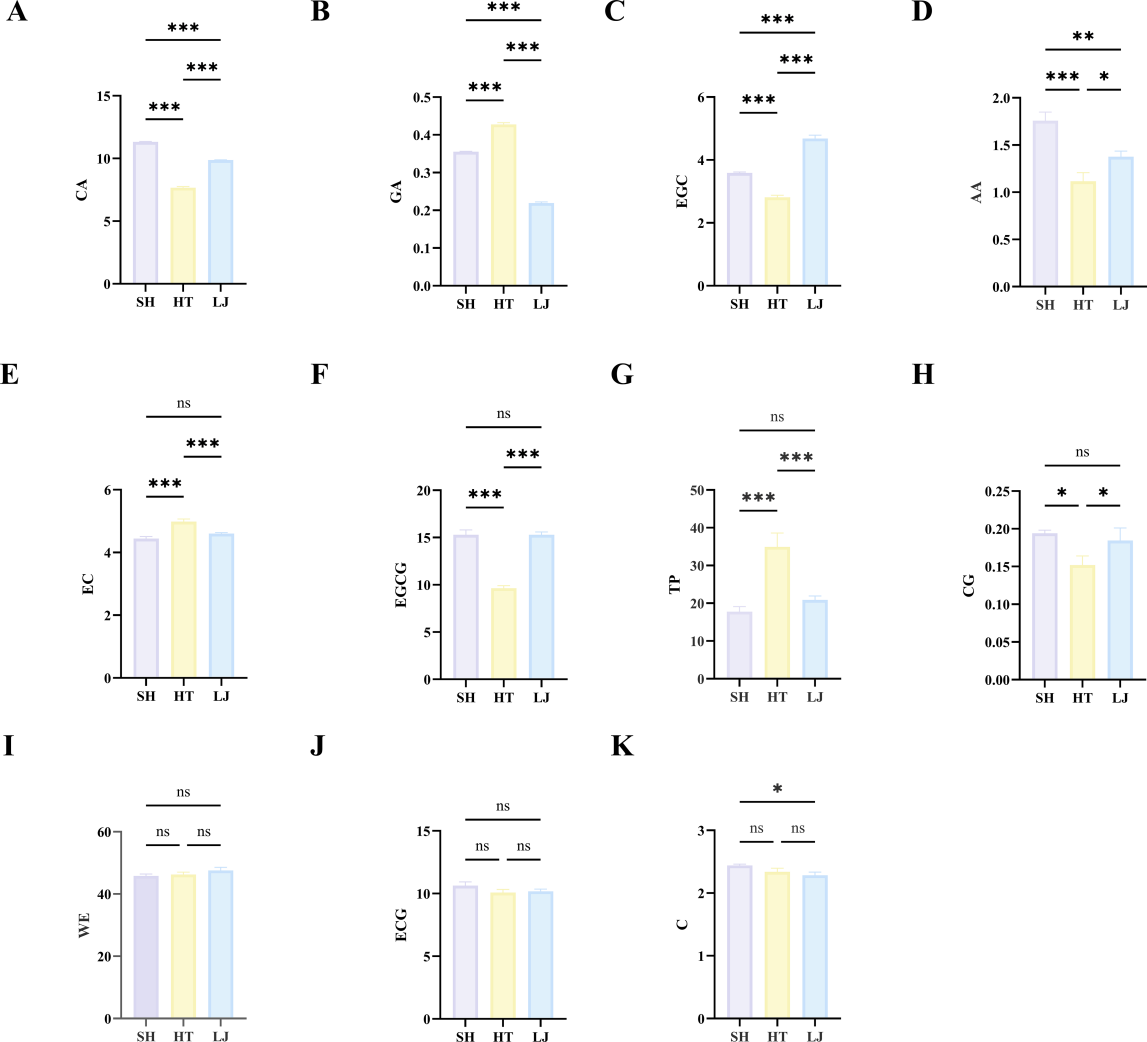
Physicochemical component contents in tea leaves. **(a)** CA (caffeine); **(b)** GA (Gallic acid); **(c)** EGC (epigallocatechin); **(d)** AA (Amino acid); **(e)** EC (epicatechin); **(f)** EGCG (epigallocatechin gallate); **(g)** TP (total polyphenols); **(h)** CG (catechin gallate); **(i)** WE (Water extract); **(j)** ECG (epicatechin gallate); **(k)** C (catechin). * indicates significant difference vs. control group (*P* < 0.05); **P < 0.01, ***P < 0.001.

Physicochemical components are both the material basis of tea flavor and key quality indicators (Yang et al., 2025). Analysis of the physicochemical components in tea samples from different production regions indicates significant differences in total polyphenols (TP), catechins (epicatechin (EC), epigallocatechin (EGC), epigallocatechin gallate (EGCG), catechin gallate (CG)), caffeine (CA), and amino acids (AA) among tea samples from different production regions. Total polyphenols (TP), key contributors to the astringency and bitterness of tea infusion, were significantly higher in HT than in SH and LJ (*P* < 0.05). Notably, major tea catechins (EGCG, EGC), key drivers of tea liquor astringency, had significantly lower levels in HT than in SH and LJ (Ye et al., 2022). Amino acids (AA), which balance the astringency and bitterness of tea infusion while enhancing umami taste, showed the highest levels in SH and the lowest in HT (*P* < 0.05) (**Figure 2**) (Zhang et al., 2020). In summary, significant differences in tea quality were observed among different production regions, and these variations were jointly associated with changes in catechins, amino acids, and caffeine, among which catechins showed particularly pronounced regional differences.

### 3.2 Soil physicochemical properties of three core production regions

**Figure 3:**
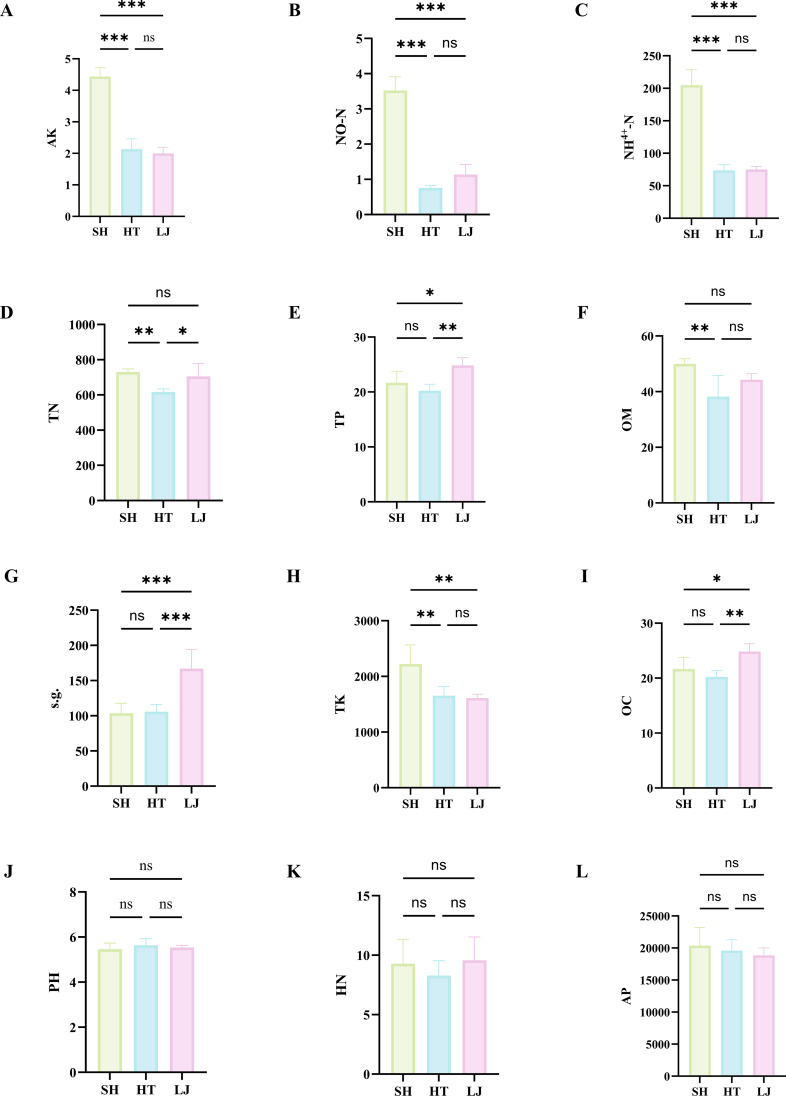
Soil physicochemical property contents. **(a)** AK (available potassium); **(b)** NO^2−^-N (nitrite nitrogen); **(c)** NH 4^+^ -N (ammonium nitrogen); **(d)** TN (total nitrogen); **(e)** TP (total phosphorus); **(f)** OM (organic matter); **(g)** SG (specific gravity); **(h)** TK (total potassium); **(i)** OC (organic carbon); **(j)** pH; **(k)** HN (hydrolyzable nitrogen); **(l)** AP (available phosphorus). * indicates significant difference vs. control group (*P* < 0.05); **P < 0.01, ***P < 0.001.

Climatic and soil conditions are key determinants of tea cultivation and quality (Mondal et al., 2025). Under consistent climate, location, cultivar, and agronomic management conditions. Soil property effects on tea quality were analyzed to elucidate drivers of quality variations across production regions. Soil, as a critical growth substrate for tea plants, exerts a pronounced impact on tea quality. We first determined the soil physicochemical properties (**Figure 3**) (Jibola-Shittu et al., 2024).

Among the 12 measured soil physicochemical indicators, 9 parameters, organic carbon (OC), total potassium (TK), specific gravity (s.g.), nitrate nitrogen (NO_3_^-^-N), organic matter (OM), available potassium (AK), ammonium nitrogen (NH_4_^+^-N), total nitrogen (TN), and total phosphorus, exhibited significant differences across different production regions (*P* < 0.05). This suggests that soil physicochemical properties may be key drivers of tea quality variations among these regions. Correlation analysis revealed that total polyphenols (TP) and epicatechin (EC) in tea leaves were significantly negatively correlated with soil physicochemical components, whereas caffeine (CA) and epigallocatechin (EGC) were significantly positively correlated with these components (|r| = 0.8) (**Supplementary Figure S1**). In summary, variations in soil physicochemical properties may primarily act on the accumulation process of catechin compounds (EC, EGC) in tea leaves from different production regions.

### 3.3 Soil metabolite characteristics of three core production regions

**Figure 4:**
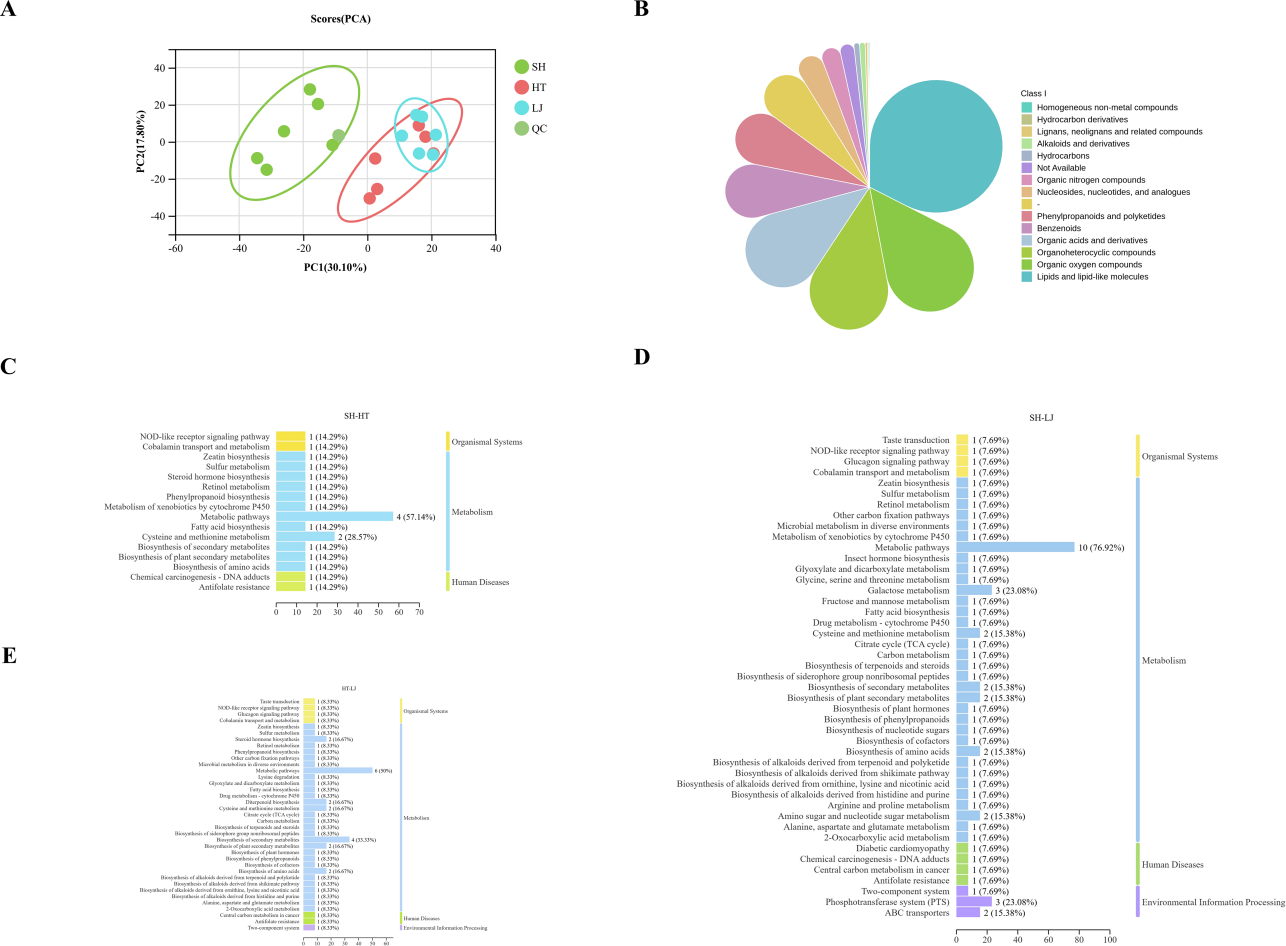
Soil metabolite analysis. **(a)** PCA of soil metabolites across different producing areas; **(b)** HMDB classification of soil metabolites across different tea-producing regions; **(c)** KEGG pathway analysis of differential metabolites between SH and LJ; **(d)** KEGG pathway analysis of differential metabolites between HT and LJ; **(e)** KEGG pathway analysis of differential metabolites between SH and HT.

Metabolites, essential products or intermediate compounds generated via enzymatic reactions, play a critical role in regulating interactions between soil microbial communities and tea plants (Zhao et al., 2022). These interactions ultimately affect plant growth and adaptability, with bioactive secondary metabolites being particularly influential (Zhuang et al., 2024). Untargeted metabolomic analysis was performed to characterize the metabolic profiles of soils from different production regions, with a total of 791 metabolites were identified and categorized into 14 classes (**Figure 4b**). The soil metabolites in SH showed significant differences from those in HT and LJ (**Figure 4a**).

We calculated P values and fold change (FC) values to identify differential metabolites among the production regions, with the screening criteria set as adjusted P values (FDR < 0.05), FC > 1.2, or FC < 0.8. Of these differential metabolites, the number identified between SH and LJ was the highest (**Supplementary Figure S2**). Differential metabolites were mainly dominated by Lipids and lipid-like molecules (28.95%), Benzenoids (21.05%) and Organoheterocyclic compounds (18.42%) (**Supplementary Figure S3**). KEGG enrichment analysis identified “Metabolic pathways” as the dominant category across all pairwise comparisons, characterized by notably high enrichment scores (**Figures 4c–e**). These results suggest that this pathway plays a central role in the metabolic remodeling observed among the three production regions, serving as a primary link to their underlying biological differences.

### 3.4 Soil microbial diversity characteristics of different production regions

To explore variations in soil microbial communities across different production regions, high-throughput sequencing was conducted separately for bacterial and fungal taxa, generating 681,369 and 711,759 high-quality sequences, respectively.

**Figure 5:**
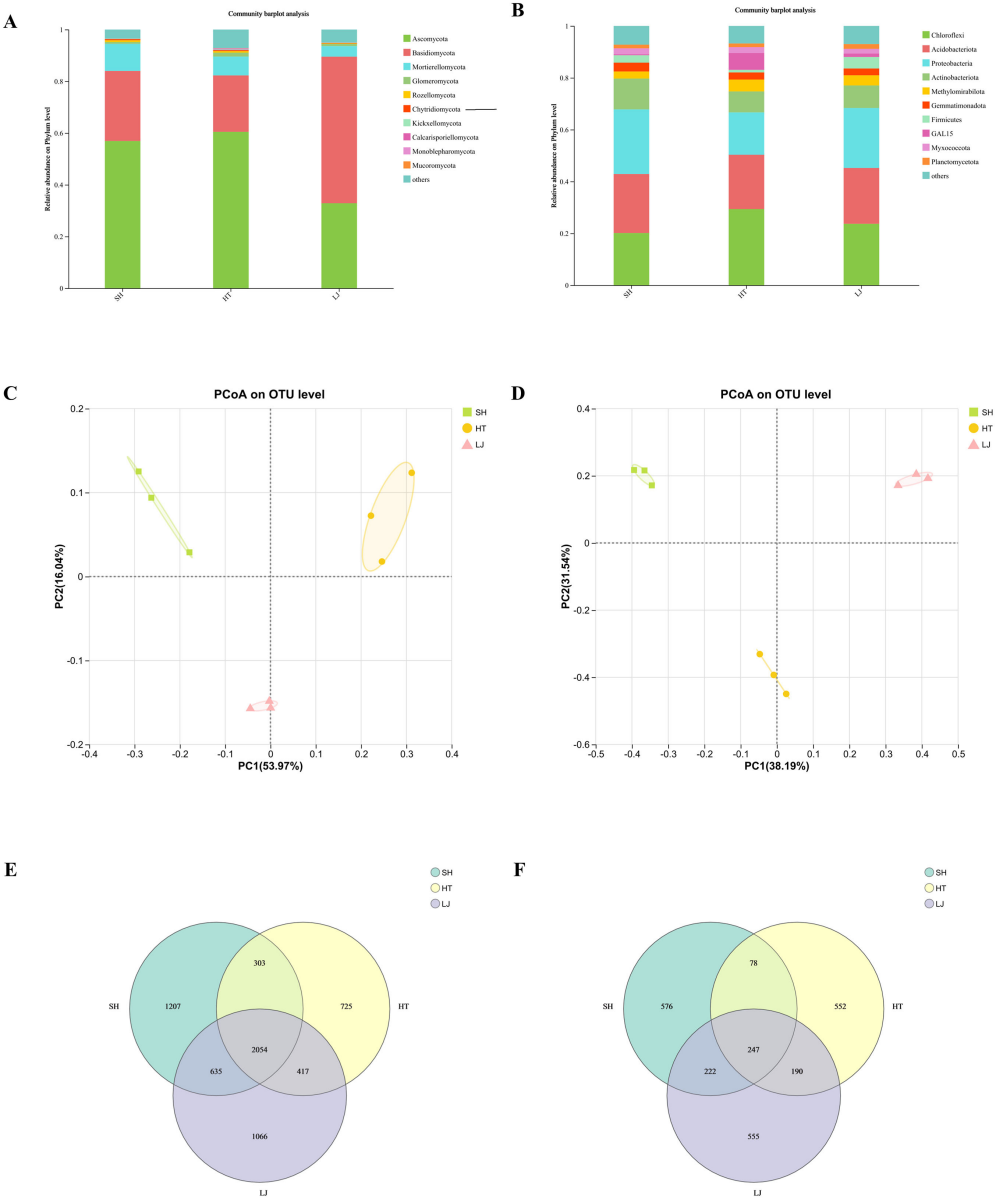
Soil microbial community composition. **(a)** Relative abundance of dominant fungal phyla in SH, HT, and LJ soils. **(b)** Relative abundance of dominant bacterial phyla in SH, HT, and LJ soils. **(c)** Principal coordinates analysis (PCoA) of fungal communities at the OTU level. **(d)** Principal coordinates analysis (PCoA) of bacterial communities at the OTU level. **(e)** Venn diagram of fungal OTUs shared among SH, HT, and LJ. **(f)** Venn diagram of bacterial OTUs shared among SH, HT, and LJ.

Annotation results revealed that *Chloroflexi*, *Acidobacteriota*, *Proteobacteria*, and *Actinobacteria* represented the dominant bacterial phyla, with variations in their relative abundances among the three production regions. In addition, the lowest relative abundance across all regions was recorded for *Actinobacteria*. Beyond the dominant phyla, the relative abundance of *GAL15* was significantly higher in the HT region compared to the SH and LJ. For fungi, *Ascomycota*, *Basidiomycota*, and *Mortierellomycota* represented the dominant phyla, with their relative abundances varying across the three production regions. *Ascomycota* had the highest relative abundance in SH and HT, emerging as the dominant phylum. *Basidiomycota* were predominantly distributed in HT, with a higher relative abundance than other fungal phyla (**Figure 5**).

**Figure 6:**
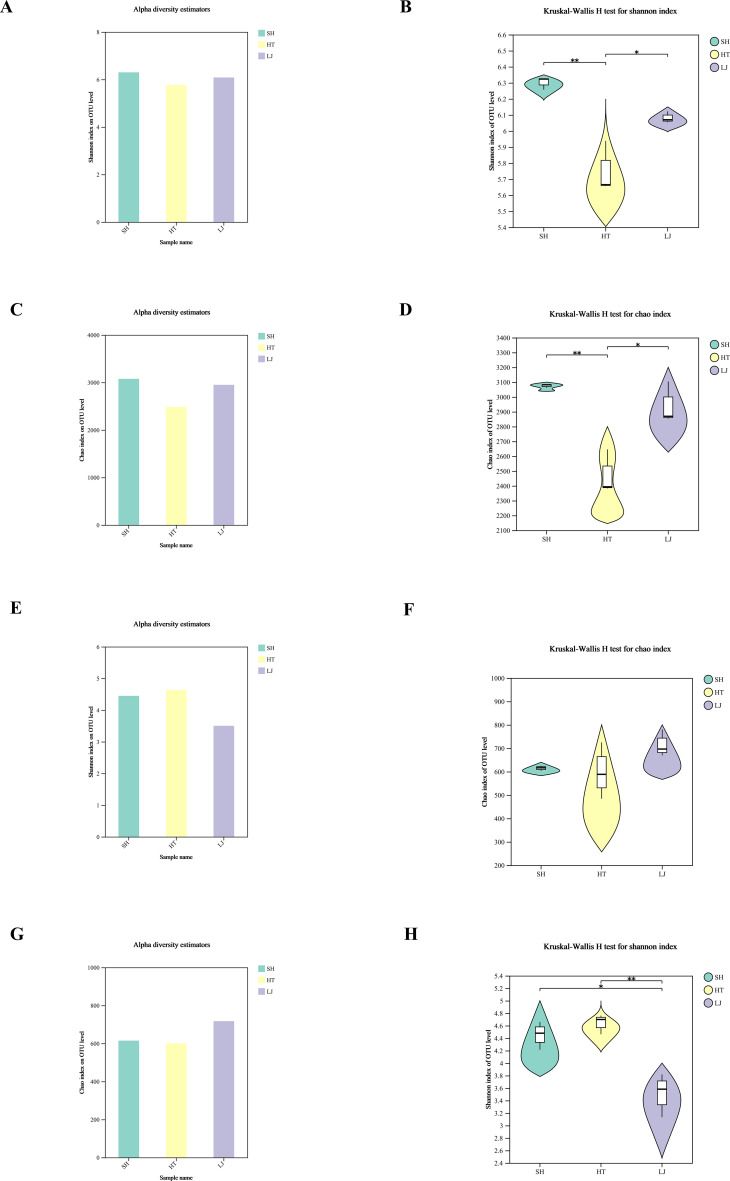
Soil microbial diversity analysis. **(a)** Bacterial Shannon index at the OTU level. **(b)** Kruskal–Wallis H test of the bacterial Shannon index. **(c)** Bacterial Chao1 index at the OTU level. **(d)** Kruskal–Wallis H test of the bacterial Chao1 index. **(e)** Fungal Shannon index at the OTU level. **(f)** Kruskal–Wallis H test of the fungal Chao1 index. **(g)** Fungal Chao1 index at the OTU level. **(h)** Kruskal–Wallis H test of the fungal Shannon index. *P < 0.05, **P < 0.01, indicating statistically significant differences in the corresponding index between groups. A P value < 0.05 is considered as the threshold for significant between-group difference.

Significant differences in bacterial diversity across different production regions were identified via alpha and beta diversity analyses, with distinct separation observed in community structures. For fungal communities, heterogeneity was detected in the Shannon and Chao1 indices, whereas beta diversity analysis revealed no significant differences among production regions. Bacterial communities showed clear regional differentiation, while fungal communities displayed heterogeneity mainly in alpha diversity rather than significant regional separation in beta diversity (**Figures 6a–h**).

**Figure 7:**
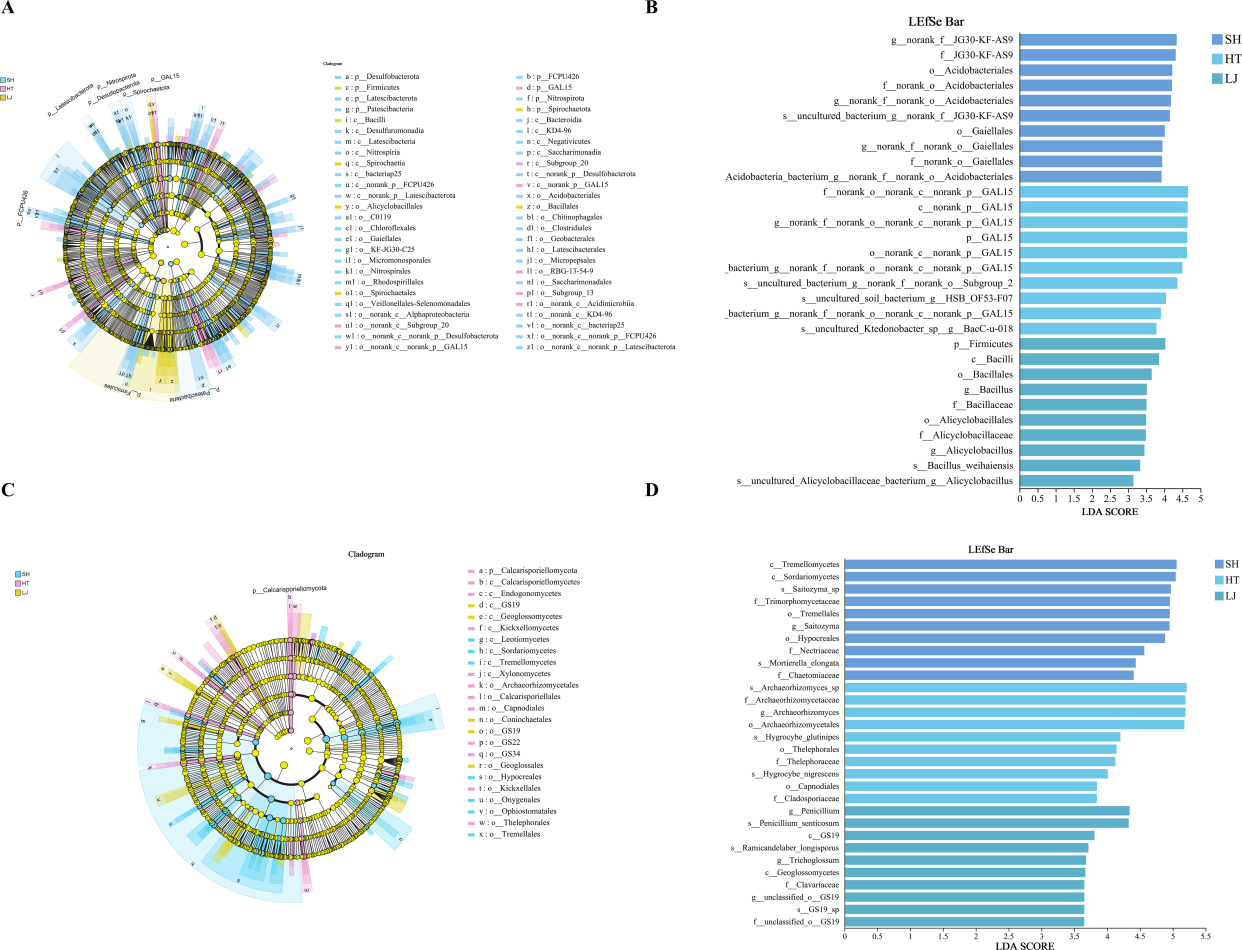
LEfSe analysis of soil microbial communities across different production regions. **(A)** LEfSe hierarchical cladogram of bacterial taxa. **(B)** LDA score histogram of bacterial taxa. **(C)** LEfSe hierarchical cladogram of fungal taxa. **(D)** LDA score histogram of fungal taxa.

LEfSe analysis was performed to screen for differentially abundant microbial community taxa in soils of different production regions (P < 0.05). At the phylum level of bacterial communities, biomarkers for SH included *p:Desulfobacterota*, *p:Patescibacteria*, *p:FCPU426*, and *p:Nitrospirota*. Biomarkers for LJ were *p:Firmicutes* and *p:Spirochaetota*. For fungal communities, *p:Calcarisporiellomycota* was identified as the biomarker for HT. At the genus level of bacterial communities, *g:Acidiphilium* and *g:Ferrovum* of HT, *g:Bacillus* and *g:Lysinibacillus* of LJ, and *g:Streptomyces* and *g:Nitrospira* of SH were significantly enriched. Bacterial communities exhibit a greater abundance of biomarkers among different production regions. These biomarkers play an important role in distinguishing soil microbial community composition among different production regions (**Figure 7**).

### 3.5 Correlation analysis among soil microbes, soil metabolites, and tea quality

**Figure 8:**
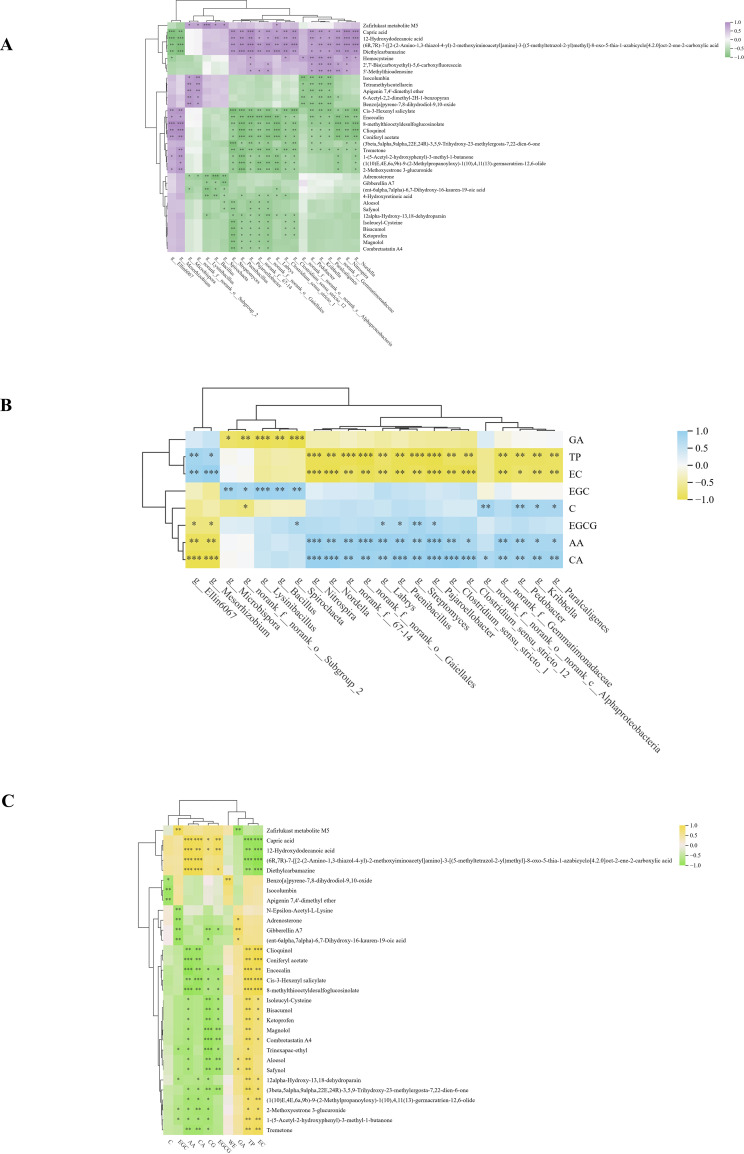
Correlation analysis of soil microorganisms, soil metabolites, and tea physicochemical components. **(a)** Correlation analysis between soil microorganisms and soil metabolites; **(b)** Correlation analysis between soil microorganisms and physicochemical components of tea leaves. **(c)** Correlation analysis between soil metabolites and tea physicochemical components.

Spearman correlation analysis was performed among differentially abundant soil bacterial taxa, soil metabolites, and tea physicochemical components across regions. The results showed that *g:Streptomyces* was significantly negatively correlated with amino acid metabolites but positively correlated with total amino acids (AA) in tea, while amino acid metabolites were negatively correlated with AA (Zhou et al., 2024). Meanwhile, *g:Bacillus* was significantly negatively correlated with the soil metabolite gibberellin A7, positively correlated with EGC, and negatively correlated with GA. In addition, gibberellin A7 was positively correlated with GA and negatively correlated with EGC (**Figure 8**). These results indicate significant correlations among differential bacterial taxa, metabolites, and tea physicochemical components.

## 4 Discussion

The unique geographical conditions of Yunnan have endowed ancient Pu-Erh tea from small production regions with distinctive characteristics, which have gained wide market recognition. However, the distinctive flavor and scarce resources have driven surging demand, leading to excessive harvesting and intensified anthropogenic disturbances. These activities have impaired the health of ancient tea plants and the biodiversity of tea gardens, thereby threatening their sustainable development (Zhang et al., 2022; Li et al., 2023). Soil serves as the core medium for tea plant growth, microorganisms constitute a key component of the soil ecosystem, and soil metabolites profoundly regulate microbial community structure and soil physicochemical properties (Li et al., 2022; Wang et al., 2024). Focusing on the core production regions of Laowu Mountain Region, this study aims to elucidate the key roles of soil microorganisms and metabolites, mediated by the soil environment, in regulating tea quality by combining 16S/ITS amplicon sequencing and untargeted liquid chromatography-tandem mass spectrometry (LC-MS/MS) metabolomics.

Firstly, determination of the physicochemical components of tea from different production regions revealed significant differences in tea quality among these regions. Climate and soil are the core environmental factors regulating tea quality (Bania et al., 2025). Given the high similarity in climate across the core production regions, we hypothesize that soil is one of the key driving factors contributing to the differences in tea quality among the regions. Nevertheless, as this study was conducted under field conditions, the influence of potential non-soil environmental heterogeneity could not be fully eliminated and should be considered when interpreting the observed associations.

Soil physicochemical heterogeneity across different production regions may reshape the composition and abundance of soil metabolites, thereby influencing tea plant growth and quality formation (Li et al., 2022; Jiang et al., 2024; Peng et al., 2025). In this study, clear differences in soil metabolic profiles were observed among regions, with SH showing distinct separation from HT and LJ, and the largest number of differential metabolites detected between SH and LJ. This pattern suggests that regional variation in soil environments is accompanied by pronounced metabolic divergence. Differential metabolites were mainly enriched in lipids and lipid-like molecules, benzenoids, and organoheterocyclic compounds. These metabolite classes are often associated with microbial activity, rhizosphere interactions, and organic matter transformation, and may therefore contribute to differences in tea plant adaptation across regions (Xie et al., 2022; Tang et al., 2024). KEGG enrichment analysis further indicated that “Metabolic pathways” was the most prominent category across all pairwise comparisons, suggesting that shifts in soil metabolic pathways may influence nutrient cycling and plant–microbe interactions, thereby contributing to regional variation in tea quality (Takénaka and Moras, 2020; Li et al., 2024).

Soil microbial communities also differed markedly among production regions, with bacterial communities showing stronger geographic differentiation than fungal communities, suggesting greater sensitivity to regional soil heterogeneity. This pattern implies that bacteria may play a more direct role in nutrient turnover and tea quality formation. Although the dominant bacterial and fungal taxa identified here are commonly found in tea plantation soils, their regional variation likely reflects differences in nutrient status, organic matter turnover, and rhizosphere processes rather than simple taxonomic shifts. Notably, Chloroflexi was particularly prominent in this study. As a widely distributed phylum involved in carbon, nitrogen, and sulfur cycling, Chloroflexi can proliferate under conditions shaped by organic fertilization and irrigation (Zhou et al., 2025). Its high abundance in the Laowu Mountain region may therefore be associated with the acidic, humid soil microenvironment and long-term organic matter accumulation. In addition, several enriched bacterial biomarkers, including *g:Streptomyces*, *g:Bacillus*, *g:Lysinibacillus*, and *g:Nitrospira*, are linked to nutrient transformation and plant–soil interactions, indicating potential functional divergence among regions (Bag et al., 2022). Compared with modern tea plantations, ancient tea gardens generally experience lower management intensity and less anthropogenic disturbance, which may favor more stable and locally adapted microbial communities. This may partly explain the pronounced regional microbial signatures observed in ancient tea garden soils and their close association with variation in tea quality (Yang et al., 2024).

Subsequent correlation analysis revealed a coordinated relationship among differentially abundant bacterial taxa, soil metabolites, and tea physicochemical components. In particular, *g:Bacillus* was significantly negatively correlated with gibberellin A7 (GA7) and GA, but positively correlated with EGC, whereas GA7 showed a positive correlation with GA and a negative correlation with EGC. These association patterns suggest that *Bacillus* may be involved in tea quality formation by influencing soil metabolic processes related to phytohormone dynamics. *Bacillus* is widely recognized as a functional genus in tea rhizosphere soils and has been reported to promote plant growth through nutrient mobilization, phytohormone regulation, and rhizosphere interaction (Saxena et al., 2020). Based on the correlation patterns observed here, the enrichment of Bacillus may be associated with reduced accumulation of GA-related metabolites in soil, which could in turn be linked to variation in tea biochemical components. In contrast, the negative relationship between GA7 and EGC suggests that changes in diterpenoid-related metabolites may be coupled with lower accumulation of certain catechin components. Although the underlying mechanism remains unclear, these findings support the possibility that soil microorganisms and metabolites are interconnected in shaping tea quality traits. It should be noted, however, that the present evidence is based on correlation analysis and does not establish direct causality. Therefore, the potential role of Bacillus in mediating the balance between hormone-related metabolites and tea quality components should be further tested using targeted metabolomics, microbial isolation, and functional validation experiments.

## 5 Conclusion

In summary, significant differences in tea quality were observed among the core production regions of the Laowu Mountain Region, with soil microorganisms and metabolites identified as key drivers. These findings support a coupled relationship among soil properties, microbial communities, metabolites, and tea quality in ancient tea gardens. They further indicate that terroir effects in ancient tea plants are shaped not only by geographic and climatic conditions, but also by belowground ecological processes involving microbial assembly and metabolic differentiation. From a practical perspective, this study provides a basis for the precise management of small tea-producing areas through soil ecological regulation, including the maintenance of soil quality, beneficial microorganisms, and region-specific metabolic functions. It also highlights the importance of conserving soil biodiversity and ecological stability in ancient tea gardens to support the sustainable utilization of Yunnan ancient tea plant resources.

## Data Availability

The datasets presented in this study can be found in online repositories. The names of the repository/repositories and accession number(s) can be found in the article/**Supplementary Material**.
