# Supplementary material for: The soil microbiome and metabolome in concert shape the flavor profile of ancient tea plants from Laowu mountain region
Source: Front Plant Sci. 2026 Apr 23;17:1797648. doi: 10.3389/fpls.2026.1797648 (PMC13149383; doi:10.3389/fpls.2026.1797648)

Supplementary Material

1 Supplement Table 1: Sample Collection Information

Tributary	Altitudes (m)	Longitude and Latitude	Sample classification			
			Soil			Tea
			Metabolize	Microbial	Physicochemical	
SH	2055.73	100°43′E, 23°52′N	01-06	01-03	01-05	01-03
HT	2117.70	100°42′E, 23°49′N	01-06	01-03	01-05	01-03
LJ	2047.32	100°44′E, 23°51′N	01-06	01-03	01-05	01-03

Note: “01-06” indicates that six soil metabolite samples were collected, numbered 01 through 06.

Table 1：Supplementary Table S1. Annotation information of key differential metabolites identified in soil samples

Metabolite	Metab ID	Library ID	KEGG Compound ID	M/Z	Retention time	Mode	Adducts	Formula	Fragmentation Score	Theoretical Fragmentation Score	Mass Error	CAS ID	RSD
(6R,7R)-7-[[2-(2-Amino-1,3-thiazol-4-yl)-2-methoxyiminoacetyl]amino]-3-[(5-methyltetrazol-2-yl)methyl]-8-oxo-5-thia-1-azabicyclo[4.2.0]oct-2-ene-2-carboxylic acid	metab_2197	HMDB0247499	-	462.0767	0.5623	pos	M+H-H2O	C16H17N9O5S2	0	47.9	1.270139322	-	0.067687344
(ent-6alpha,7alpha)-6,7-Dihydroxy-16-kauren-19-oic acid	metab_5646	HMDB0036763	C11876	315.1972	5.8379	neg	M-H2O-H	C20H30O4	0	51	1.986024928	26109-32-0	0.045759753
12alpha-Hydroxy-13,18-dehydroparain	metab_5242	HMDB0039557	-	421.1875	6.3396	neg	M+FA-H	C21H28O6	0	50.8	1.948329838	-	0.146707857
12-Hydroxydodecanoic acid	metab_5718	HMDB0002059; HMDB0304547	C08317	431.3384	5.7873	neg	2M-H	C12H24O3	0	30.8	1.450084754	505-95-3	0.032994411
2',7'-Bis(carboxyethyl)-5,6-carboxyfluorescein	metab_9	HMDB0245556	-	606.1193	0.6983	pos	M+ACN+H	C28H20O13	0	48.8	-8.679451759	-	0.050380453
5'-Methylthioadenosine	metab_3318	HMDB0001173; PW_C000910	C00170	890.2643	0.7563	neg	3M-H	C11H15N5O3S	0	36.8	3.203846267	2457-80-9	0.019624505
6-Acetyl-2,2-dimethyl-2H-1-benzopyran	metab_4004	HMDB0030816	-	247.0981	5.6935	neg	M+FA-H	C13H14O2	0	48.7	2.401430036	19013-07-1	0.023174855
8-methylthiooctyldesulfoglucosinolate	metab_7832	HMDB0304249	-	442.1571	0.6157	neg	M+FA-H	C16H31NO6S2	0	68.7	-1.019300342	-	0.023598848
Aloesol	metab_201	HMDB0035712	-	267.1225	5.2809	pos	M+CH3OH+H	C13H14O4	0	65.8	-0.687273995	94356-35-1	0.025910025
Apigenin 7,4'-dimethyl ether	metab_5989	HMDB0132454; LMPK12111029	C10019	297.0775	5.6444	neg	M-H	C17H14O5	0	48.2	2.104352081	5128-44-9	0.009143629
Benzo[a]pyrene-7,8-dihydrodiol-9,10-oxide	metab_7844	HMDB0062470	C14853	663.2006	0.6348	neg	2M+Hac-H	C20H14O3	0	33.3	-3.055058401	55097-80-8;58917-67-2	0.011773037
Bisacumol	metab_385	HMDB0038511	-	459.3243	6.0716	pos	2M+Na	C15H22O	0	40.5	2.272390522	120710-98-7	0.155679332
Capric acid	metab_4070	HMDB0000511; PW_C000391; LMFA01010010	C01571	389.2917	5.7403	neg	2M+FA-H	C10H20O2	0	31.4	2.39282615	334-48-5	0.017338549
Cephaloglycin	metab_2156	HMDB0014827	C13440	370.0835	0.5943	pos	M+H-2H2O	C18H19N3O6S	0	35.4	-5.072213164	612516	0.017248056
Cis-3-Hexenyl salicylate	metab_5842	HMDB0061942; HMDB0061823	-	219.1028	5.3886	neg	M-H2O-H, M-H	C13H16O3	0	39.9	0.765775739	-	0.004456275
Clioquinol	metab_5430	HMDB0250327	-	608.8135	6.0469	neg	2M-H	C9H5ClINO	0	57.4	-0.092303356	-	0.051904765
Combretastatin A4	metab_6108	HMDB0253622; HMDB0250413	C20268	315.1246	5.5284	neg	M-H	C18H20O5	0	69.1	2.686513983	117048-59-6	0.016911075
Cyclopamine	metab_1124	HMDB0250664	C10798	453.3435	6.3898	pos	M+ACN+H	C27H41NO2	0	70	-9.816781722	4449-51-8	0.016607123
Diethylcarbamazine	metab_5588	HMDB0014849; MJDBOTE0000224	C07968	443.3383	5.895	neg	2M+FA-H	C10H21N3O	0	35.8	7.912524939	90-89-1	0.073208905
Encecalin	metab_1538	-	C09005	233.1173	5.8087	pos	M+H	C14H16O3	88.2	0	0.369855412	20628-09-5	0.003639919
Gibberellin A7	metab_3907	HMDB0303475; LMPR0104170024	C11867	311.1297	5.5832	neg	M-H2O-H	C19H22O5	0	51.2	2.380453162	510-75-8	0.018486983
Grossamide	metab_6030	HMDB0040370	-	623.2408	5.6076	neg	M-H	C36H36N2O8	0	56.7	1.458032255	80510-06-1	0.019351743

2 Supplementary Figure 1 :Correlation analysis of tea physicochemical components and soil physicochemical properties

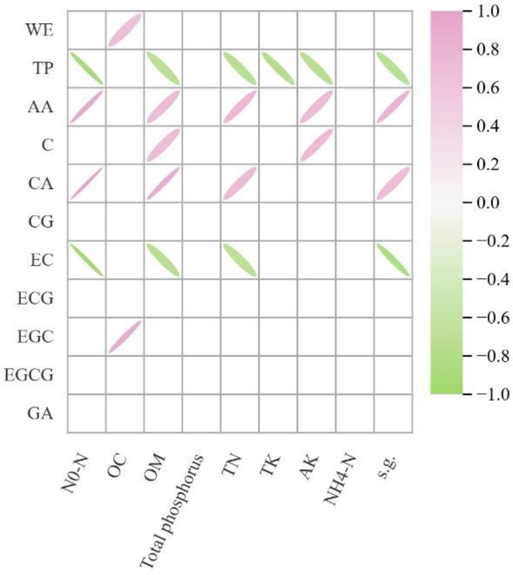

3 Supplementary Figure 2: Statistical comparison of differential metabolite abundance

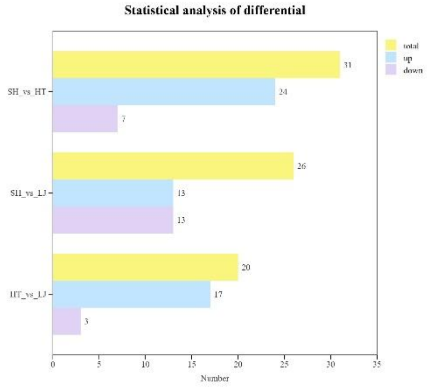

4 Supplementary Figure 3: Composition of differential soil metabolites across production regions

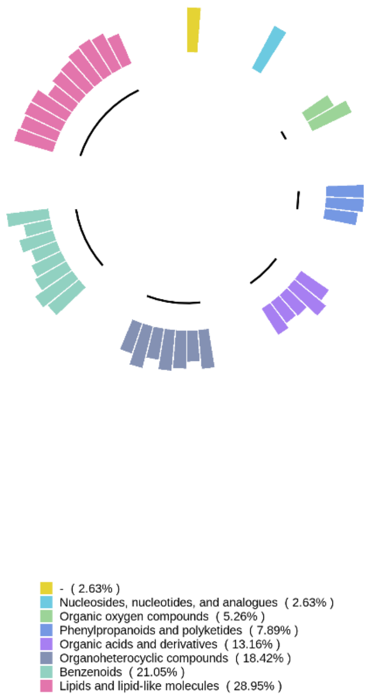

Supplement: Supplementary file 1 [file DataSheet1.pdf]
